# Visual Performance and Satisfaction of Extended Depth of Focus Contact Lenses in Presbyopic Subjects

**DOI:** 10.3390/jcm14030818

**Published:** 2025-01-26

**Authors:** Kazutaka Kamiya, Shota Tokuda, Tomoko Kaida, Shizuka Higashi, Midori Hashiguchi, Eriko Kanaya, Kazunori Miyata

**Affiliations:** 1 Visual Physiology, School of Allied Health Sciences, Kitasato University, Sagamihara 2520373, Japan; 2 Department of Ophthalmology, Miyata Eye Hospital, Miyazaki 8850051, Japan; tokuda@miyata-med.ne.jp (S.T.); kaida@miyata-med.ne.jp (T.K.); s-higashi@miyata-med.ne.jp (S.H.); hashiguchi@miyata-med.ne.jp (M.H.); kanaya@miyata-med.ne.jp (E.K.); miyata@miyata-med.ne.jp (K.M.)

**Keywords:** extended-depth-of-focus contact lens, presbyopia, visual acuity, higher-order aberrations, satisfaction

## Abstract

**Background/Objectives:** The objective was to assess visual performance and the overall satisfaction of extended-depth-of-focus (EDOF) contact lenses (CLs) in a presbyopic population. **Methods:** We prospectively investigated visual acuity at all distances (0.3, 0.4, 0.5, 0.7, 1, and 5 m), higher-order aberrations (HOAs), contrast sensitivity function, and overall satisfaction score, before and during EDOF CL wear in 42 eyes of 21 presbyopic subjects (1 man and 20 women). **Results:** Binocular visual acuity at 0.3, 0.5, 0.7, 1, 2, 3, and 5 m when wearing EDOF CLs was 0.01 ± 0.14, −0.08 ± 0.02, −0.08 ± 0.02, −0.08 ± 0.00, −0.08 ± 0.00, −0.08 ± 0.02, and −0.07 ± 0.02, respectively. We found a significant improvement at near to intermediate distances (0.3, and 0.5 m), but no significant change at intermediate to far distances (0.7, 1, 2, 3, and 5 m) between before and during CL wear. The area under the log contrast sensitivity function was not significantly changed under photopic nor mesopic conditions (*p* = 0.099, and *p* = 0.689). Ocular third-order aberrations, fourth-order aberrations, or total HOAs for a 4 mm pupil significantly increased. The overall satisfaction score significantly improved by wearing EDOF CLs. **Conclusions:** EDOF CLs significantly improved visual acuity at near to intermediate distances, while maintaining far vision, and the effect of contrast sensitivity was minimal, suggesting the viability of presbyopic correction in such candidates.

## 1. Introduction

The global presbyopic population has been estimated to have grown to one billion, and thus the demand for presbyopic correction has steadily grown over time [1]. Multifocal contact lenses (CLs) have demonstrated significant efficacy in facilitating optimal visual acuity across various distances, ranging from near to far, thereby obviating the need for spectacles among presbyopic subjects. As a result of these favorable outcomes, multifocal CLs have garnered widespread acknowledgment as one of the foremost non-surgical viable alternatives for addressing presbyopic correction [2,3,4]. In fact, a wide variety of multifocal CL designs have become available in daily practice, but these multifocal CLs are dependent on the effective optical diameter, illuminance environment, as well as lens centration and movement, and thus are susceptible for the effect of pupil variations, illumination levels, and lens fitting, resulting in a deterioration in visual quality. In actuality, evidence suggests that the design of multifocal contact lenses (CL) plays a role in influencing the discontinuation of CL wear [5].

Extended depth of focus (EDOF) CLs have been developed by the Brien Holden Vision Institute and have made significant strides in development and are now accessible in the commercial market, notably becoming available in Japan in recent years. Theoretically, EDOF CLs hold several potential advantages over traditional multifocal CLs. These include reduced reliance on pupil size variations, diminished sensitivity to CL movement, and mitigated declines in contrast sensitivity. Indeed, reports have highlighted the efficacy of EDOF CLs in facilitating good near to intermediate vision without compromising far vision, thereby underscoring their potential as a viable option for presbyopic correction [6,7,8,9,10,11]. However, these previous studies were rather limited and mostly conducted by the same research group using prototype CLs, and thus this should be evaluated by many research groups using commercially available CLs for the further prevalence of this novel CL in a clinical setting. Moreover, detailed all-distance visual acuities have not so far been investigated for presbyopic healthy subjects. This will give us essential insights into understanding the patient-derived characteristics of EDOF CLs in such a population. The objective of the current study is to conduct a prospective evaluation of the comprehensive visual performance and satisfaction levels associated with EDOF CLs in presbyopic subjects.

## 2. Materials and Methods

### 2.1. Study Population

This prospective observational study involved a comprehensive examination of 42 eyes of 21 consecutive presbyopic subjects (1 man and 20 women, mean age ± standard deviation: 50.6 ± 7.5 years). Eyes with a corrected visual acuity of <0 logMAR, eyes with a manifest astigmatism of ≥1.25 diopter (D), and eyes with any history of ocular surgery, ocular trauma, or other concomitant eye diseases, were excluded from the study. Approval for the study was diligently obtained from the Institutional Review Board at Miyata Eye Hospital (identifier; CS-394 and date of approval 27 April 2023) and is compliant with the principles outlined in the Declaration of Helsinki. Prior to their participation, all subjects were provided with comprehensive explanations regarding the study’s objectives, methodologies, potential implications, and the possible consequences. Subsequently, informed consent was obtained from each participant, ensuring their understanding and voluntary agreement to take part in the research endeavor.

### 2.2. Extended-Depth-of-Focus Contact Lens Prescription

We used a commercially available EDOF CL (1dayPure EDOF^TM^, SEED Co., Ltd., Tokyo, Japan) in the current study. This particular CL features a non-monotonic, aperiodic, non-diffractive, non-aspheric, refractive power profile across the optic zone. The EDOF is achieved by the deliberate manipulation of multiple modes of spherical aberration terms [12,13]. Notably, the lens offers 3 different depth of focus profiles (+0.75 D, +1.50 D, and +2.25 D) for near vision in the central region, coupled with a base curve of 8.4 mm, and a lens diameter of 14.2 mm. During an initial trial phase, the lens power was targeted at emmetropia with the lowest depth of focus (+0.75 D), based on the manufacturer’s instructions. According to the manufacturer’s instructions, the spherical power and the depth of focus were adjusted after a preliminary evaluation and were determined based on patient preferences of far to near binocular vision. Contact lens fitting was confirmed by experienced ophthalmologists (TK and EK). Subjects were instructed to wear these CLs daily for a duration of 2 to 4 weeks as part of the study protocol.

### 2.3. Assessment of Visual Performance

We conducted a comprehensive quantitative evaluation of various visual parameters in the study population, encompassing visual acuity at all distances (0.3, 0.5, 0.7, 1, 2, 3, and 5 m) using an all-distance vision tester (AS-15, Kowa, Nagoya, Japan) before and during EDOF CL wear. Additionally, higher-order aberrations (HOAs), contrast sensitivity, and patient satisfaction were assessed both before and during the EDOF CL wear in the study population. To analyze ocular HOAs, measurements were taken for 4 and 6 mm pupils with a Hartmann–Shack aberrometer (KR-1W, Topcon, Tokyo, Japan). Total ocular HOAs were then calculated as the root-mean-square of the third- and fourth-order coefficients, providing insights into the overall optical quality of the eyes. Moreover, the contrast sensitivity function was evaluated using a functional vision analyzer (Optec 6500, Stereo Optical, Chicago, IL, USA) under both photopic (85 cd/m^2^) and mesopic (3 cd/m^2^) lighting conditions. The subjects were encouraged to respond, but not to guess. A forced choice and a strict time limit were not employed during the test. We determined the area under the log contrast sensitivity function (AULCSF) by the CS data. The AULCSF was derived from the obtained contrast sensitivity data, using established methodologies [14]. In brief, the log of contrast sensitivity was plotted as a function of log spatial frequency, and third-order polynomials were fitted to the data. The fitted function was integrated, and the resultant value was expressed as the AULCSF. Furthermore, overall satisfaction levels were quantified using visual analog scale (VAS) scores ranging from 0 (indicating no satisfaction) to 100 (representing maximum satisfaction). All examinations were carried out by experienced ophthalmic technicians, ensuring the accuracy and reliability of the gathered data throughout the study process.

### 2.4. Statistical Analysis

Statistical analyses were performed utilizing a statistical software package (Bellcurve for Excel, ver.4.04; Social Survey Research Information Co, Ltd., Tokyo, Japan). Because all data did not fulfill the criteria for normal distribution by the Kolmogorov–Smirnov test, the Wilcoxon signed-rank test was employed to compare the two groups before and during CL wear. The results are described as mean ± standard deviation, and a value of *p* < 0.05 was deemed statistically significant.

## 3. Results

**Figure 1:**
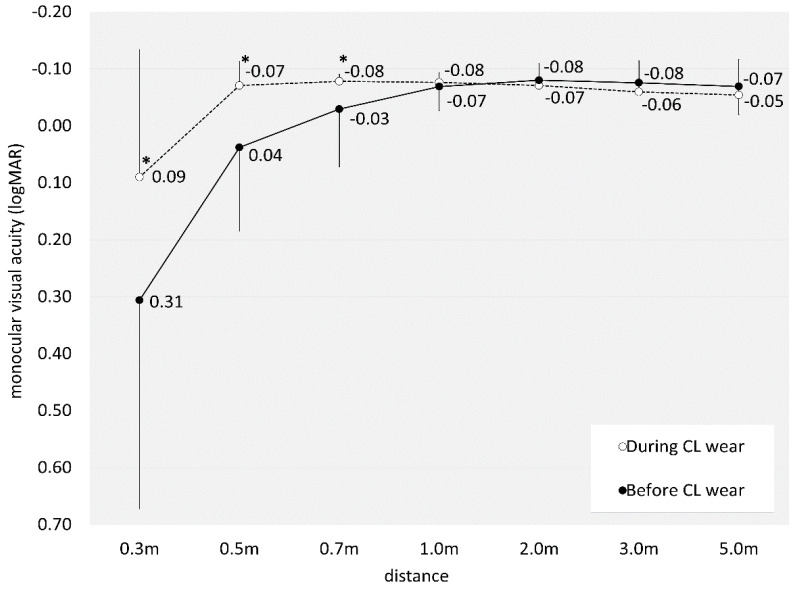
Graph showing monocular visual acuity at all distances (0.3, 0.5, 0.7, 1, 2, 3, and 5 m) before and during extended-depth-of-focus contact lens wear. We found a significant improvement at near to intermediate distances (0.3, 0.5, and 0.7 m). * indicates *p* < 0.05.

**Figure 2:**
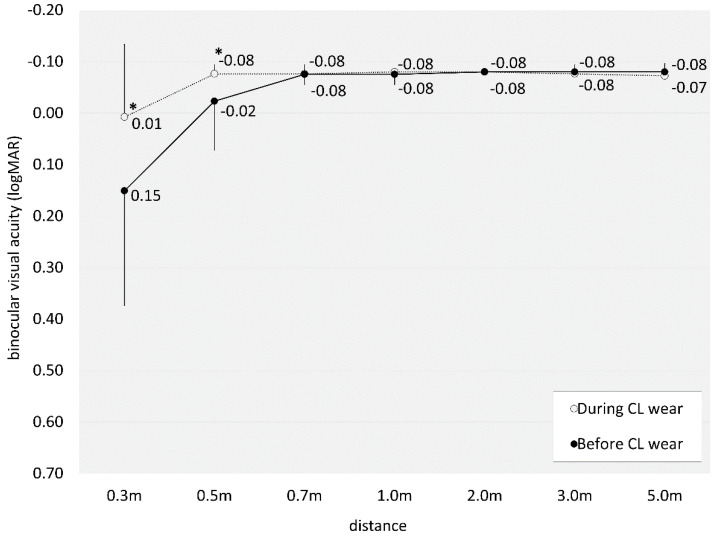
Graph showing binocular visual acuity at all distances (0.3, 0.5, 0.7, 1, 2, 3, and 5 m) before and during extended-depth-of-focus contact lens wear. We found a significant improvement at near to intermediate distances (0.3, and 0.5 m). * indicates *p* < 0.05.

Table 1 represents a breakdown of the demographic characteristics observed within the study cohort. The numbers of eyes fitted with a depth-of-focus designation of +0.75, +1.50, and +2.25 D were 30 (71%), 12 (29%), and 0 (0%) eyes, respectively. Notably, neither CL-related nor non-related complications, including dry eye syndrome, were reported in any case throughout the entire observation period. Assessment of monocular visual acuity at 0.3, 0.5, 0.7, 1, 2, 3, and 5 m during EDOF CL wear was 0.09 ± 0.22, −0.07 ± 0.04, −0.08 ± 0.01, −0.08 ± 0.02, −0.07 ± 0.04, −0.06 ± 0.05, and −0.05 ± 0.06, respectively. There was a significant improvement at near to intermediate distances (0.3, 0.5, and 0.7 m) (Wilcoxon signed-rank test, *p* < 0.001, *p* < 0.001, and *p* = 0.003), but no significant change at intermediate to far distances (1, 2, 3, and 5 m) (*p* = 0.480, *p* = 0.180, *p* = 0.070, and *p* = 0.393) between before and during CL wear (Figure 1). Binocular visual acuity at 0.3, 0.5, 0.7, 1, 2, 3, and 5 m during EDOF CL wear was 0.01 ± 0.14, −0.08 ± 0.02, −0.08 ± 0.02, −0.08 ± 0.00, −0.08 ± 0.00, −0.08 ± 0.02, and −0.07 ± 0.02, respectively. There was a significant improvement at near to intermediate distances (0.3, and 0.5 m) (*p* = 0.011, and *p* = 0.026), but no significant change at intermediate to far distances (0.7, 1, 2, 3, and 5 m) (*p* = 1.000, *p* = 0.317, *p* = 1.000, *p* = 0.317, and *p* = 0.157) by EDOF wear (Figure 2).

**Figure 3:**
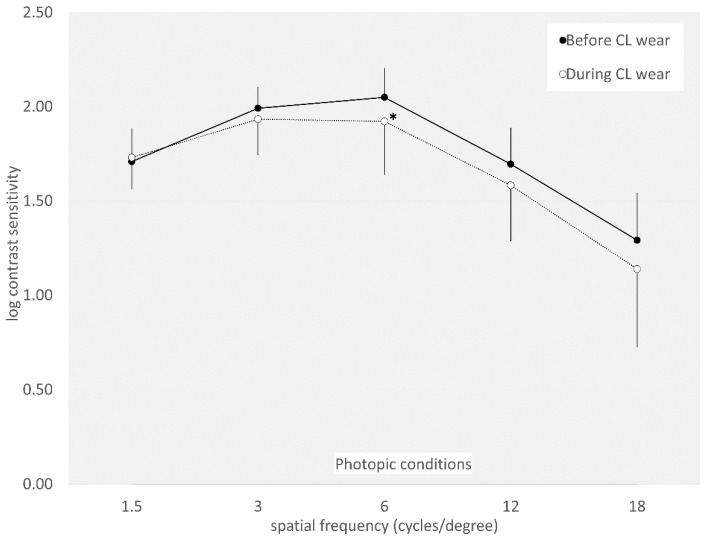
Graph showing the contrast sensitivity function before and during extended-depth-of-focus contact lens wear under photopic conditions. We found no significant changes at any spatial frequencies, except for at six cycles/degrees, under photopic conditions. * indicates *p* < 0.05.

**Figure 4:**
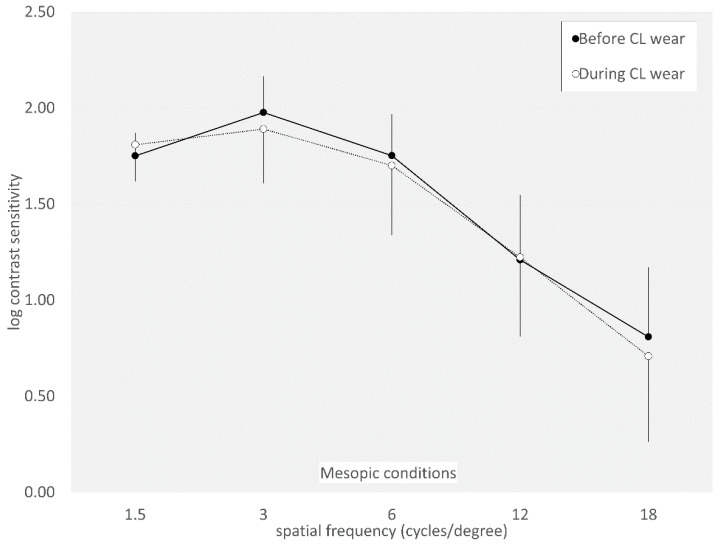
Graph showing contrast sensitivity function before and during extended-depth-of-focus contact lens wear under mesopic conditions. We found no significant changes at any spatial frequencies under mesopic conditions.

The AULCSF did not significantly change from 2.00 ± 0.12 to 1.91 ± 0.22 under photopic conditions (*p* = 0.099). It also did not significantly change from 1.76 ± 0.20 to 1.72 ± 0.31 under mesopic conditions (*p* = 0.689). Log contrast sensitivity did not significantly change at any spatial frequencies, except for at six cycles/degrees (*p* = 0.042) under photopic conditions (Figure 3 and Figure 4).

**Figure 5:**
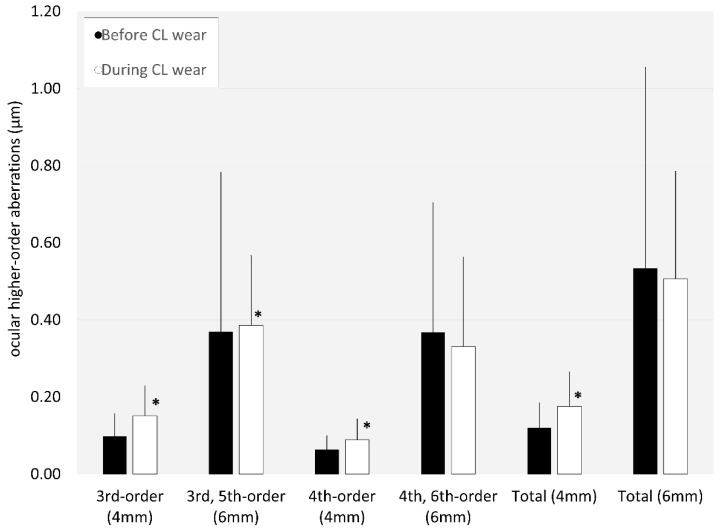
Graph showing ocular higher-order aberrations before and during extended-depth-of-focus contact lens wear. We found significant increases in third- and fourth-order aberrations, and total higher-order aberrations for a 4 mm pupil, and third- and fifth-order aberrations for a 6 mm pupil. * indicates *p* < 0.05.

Ocular higher-order aberrations significantly increased during CL wear, except for fourth-order and sixth-order or total HOAs for a 6 mm pupil (*p* = 0.563, or *p* = 0.615) (Figure 5). Additionally, there was a significant improvement in the overall satisfaction score from 37.9 ± 31.3 to 65.7 ± 23.0 during EDOF CL wear (*p* = 0.008).

## 4. Discussion

The findings of our current study indicate that EDOF CLs provided a significantly enhanced visual performance spanning from near to intermediate distances, consequently resulting in higher patient satisfaction levels. However, this improvement was accompanied by a slight decrease in contrast sensitivity function in this presbyopic population. We believe that this information will be helpful for patients who do not want to wear eyeglasses, or to receive surgical interventions such as multifocal intraocular lens implantation, in presbyopic subjects. It is worth noting that so far, there has been only one clinical study on the visual performance of this commercially available EDOF CL, albeit restricted to being focused on a pseudophakic population [11]. Hiraoka et al. [11] underscored that EDOF CLs exhibited significant enhancements in vision across near to intermediate distances even among patients undergoing monofocal intraocular lens implantation. Our findings of binocular visual acuity were slightly better than those previous findings, possibly due to the age differences in the study populations. Their conclusions, combined with our own, suggest the broad applicability of this novel EDOF CL, not only for presbyopic phakic individuals, but also for pseudophakic patients in a clinical setting. With regard to the prototype of the EDOF CL, Tilla et al. [6,7] and Bakaraju et al. [8] showed, in a prospective, randomized, clinical trial, that it provided better intermediate and near vision than other commercially available multifocal CLs without compromising far vision. Sha et al. [9] demonstrated that EDOF CLs provided subjectively better outcomes than commonly used aspheric, center-near design multifocal CLs in terms of vision clarity at near to intermediate distances, an overall lack of ghosting, vision stability, and overall vision satisfaction. Martínez-Alberquilla et al. [10] demonstrated that both EDOF and other multifocal CLs offered good visual quality without compromising the ocular surface integrity and provided similar symptomatology levels for presbyopic patients. These observations align with those of our current investigation on EDOF CLs. We observed that there were no significant decreases in contrast sensitivity function, with the exception being at six cycles/degrees under photopic conditions. Notably, contrast sensitivity function remained within the confines of the normal range throughout the duration of EDOF CL wear. Interestingly, the decline in contrast sensitivity function under mesopic light conditions appeared to be lower compared to that under photopic light conditions in this study population, as evidenced by the corresponding AULCSF values. This phenomenon could potentially be attributed to the reduced dependency on pupil diameter and illumination levels of the EDOF CL in comparison with other multifocal CLs. In line with our observations, Hiraoka et al. [11] also reported that contrast sensitivity slightly declined at the middle to high spatial frequencies among eyes that had undergone cataract surgery. We assume that this discrepancy was possibly due to the differences in the additional powers of the EDOF CL (low to middle vs. middle to high). Additionally, our analysis revealed that the change in distance visual acuity was minimal and non-significant during EDOF CL wear. This characteristic could prove useful for presbyopic patients who wish to obtain good visual outcomes especially at a far distance. We assume that this EDOF CL can be applied for presbyopic patients, especially those who wish to frequently drive a car at night. Furthermore, Novillo-Díaz et al. [5] demonstrated that poor far vision was the most common cause for multifocal CL discontinuation. Based on our findings that the effect on far vision was essentially minimal among multifocal CLs, it is plausible that this EDOF CL may also work well for sustained use in everyday activities. Several limitations should be acknowledged in the context of this study. Firstly, the sample size utilized in this study was not large, and there existed a gender bias within the study population. Such factors may influence the generalizability of our findings. Secondly, we did not conduct an assessment of the long-term visual outcomes and tolerability among wearers of EDOF CLs. Therefore, a comprehensive long-term study involving a larger and more diverse cohort of patients is warranted to validate the authenticity and robustness of our results. Such endeavors would enhance the reliability and applicability of our findings within clinical settings. Thirdly, we did not evaluate the quality of life or dry eye symptomatology survey in the current study. Although we found a significant improvement in overall patient satisfaction, it has been demonstrated that patient comfort during CL wear plays a pivotal role in successful CL wear [15,16].

## 5. Conclusions

In summary, the outcomes of our study may support the view that EDOF CLs are effective for obtaining satisfactory vision across the entire near-to-far range, consequently leading to higher levels of overall satisfaction, notwithstanding a marginal decline in contrast sensitivity function. Based on our findings, we believe that EDOF CLs are one of the feasible options for presbyopic correction, particularly when patients prioritize far vision even when wearing presbyopic CLs.

## Figures and Tables

**Table 1:** Demographics of the study population.

Patient Demographics	
Age	50.6 ± 7.5 years (range, 40 to 64 years)
Gender	Male:Female = 1:20
Corrected visual acuity (logMAR)	−0.17 ± 0.03 (range, −0.08 to −0.18)
Manifest spherical equivalent	−1.99 ± 2.49 D (range, −7.00 to 0.88 D)
Mean astigmatism	0.52 ± 0.35 D (range, 0 to 1.00 D)

logMAR = logarithm of the minimal angle of resolution; D = diopter.

## Data Availability

The data supporting the conclusions of this article will be made available by the corresponding author, K.K., upon reasonable request.

## Abbreviations

CL, contact lens; EDOF, extended depth of focus; D, diopter; HOAs, higher-order aberrations; AULCSF, area under the log contrast sensitivity function; VAS, visual analog system; logMAR, logarithm of the minimal angle of resolution.
